# Oxidation of Phe454 in the Gating Segment Inactivates *Trametes multicolor* Pyranose Oxidase during Substrate Turnover

**DOI:** 10.1371/journal.pone.0148108

**Published:** 2016-02-01

**Authors:** Petr Halada, Dagmar Brugger, Jindrich Volc, Clemens K. Peterbauer, Christian Leitner, Dietmar Haltrich

**Affiliations:** 1 Institute of Microbiology of the ASCR, v.v.i., Vídeňská 1083, Prague, Czech Republic; 2 Food Biotechnology Laboratory, BOKU—University of Natural Resources and Life Sciences Vienna, Vienna, Austria; 3 Doctoral Programme BioToP–Molecular Biotechnology of Proteins, BOKU—University of Natural Resources and Life Sciences Vienna, Vienna, Austria; USDA Forest Service, UNITED STATES

## Abstract

The flavin-dependent enzyme pyranose oxidase catalyses the oxidation of several pyranose sugars at position C-2. In a second reaction step, oxygen is reduced to hydrogen peroxide. POx is of interest for biocatalytic carbohydrate oxidations, yet it was found that the enzyme is rapidly inactivated under turnover conditions. We studied pyranose oxidase from *Trametes multicolor* (*Tm*POx) inactivated either during glucose oxidation or by exogenous hydrogen peroxide using mass spectrometry. MALDI-MS experiments of proteolytic fragments of inactivated *Tm*POx showed several peptides with a mass increase of 16 or 32 Da indicating oxidation of certain amino acids. Most of these fragments contain at least one methionine residue, which most likely is oxidised by hydrogen peroxide. One peptide fragment that did not contain any amino acid residue that is likely to be oxidised by hydrogen peroxide (DAFSYGAVQQSIDSR) was studied in detail by LC-ESI-MS/MS, which showed a +16 Da mass increase for Phe454. We propose that oxidation of Phe454, which is located at the flexible active-site loop of *Tm*POx, is the first and main step in the inactivation of *Tm*POx by hydrogen peroxide. Oxidation of methionine residues might then further contribute to the complete inactivation of the enzyme.

## Introduction

Pyranose oxidase (other names are glucose 2-oxidase or pyranose 2-oxidase, POx; systematic name pyranose:oxygen 2-oxidoreductase; EC 1.1.3.10) is a ~270 kDa, 8*α*-(*N*3)-histidyl flavinylated, homotetrameric flavin-dependent oxidase. POx is a member of the glucose-methanol-choline (GMC) family of FAD-dependent oxidoreductases, and catalyses the oxidation of several pyranose sugars at position C-2 to yield the corresponding 2-ketosugars [1–5]. In accordance with other flavin-dependent oxidoreductases POx shows a reaction mechanism consisting of two half-reactions [6,7]. In the reductive half-reaction an aldopyranose is oxidised at position C-2 to yield a 2-ketoaldose (aldos-2-ulose), accompanied by electron transfer to FAD yielding the reduced flavin, FADH_2_ (reaction 1). During the subsequent oxidative half-reaction FADH_2_ is re-oxidised by the second substrate oxygen, yielding the oxidised prosthetic group and H_2_O_2_ (reaction 2), or by an alternative electron acceptor, which can include either two-electron acceptors such as benzoquinone (reaction 3) or one-electron acceptors such as chelated metal ions [8–10]. Pyranose oxidase, and especially the enzyme from the fungus *Trametes multicolor* (*Tm*POx), has been studied in detail with respect to its structure [3,11,12] and its reaction mechanism [13,14].

$$\text{FAD}+\text{aldopyranose}\;\to \;{\text{FADH}}_{2}+\;2-\text{keto}-\text{aldopyranose}$$ (1)

$${\text{FADH}}_{2}+{\text{O}}_{2}\;\to \;\text{FAD}+\;{\text{H}}_{2}{\text{O}}_{2}$$ (2)

$${\text{FADH}}_{2}+\text{benzoquinone}\;\to \;\text{FAD}+\text{hydroquinone}$$ (3)

POx has become an attractive biocatalyst in biotransformations of carbohydrates as it can be used to synthesize various building blocks in synthetic carbohydrate chemistry and rare sugars [15,16]. In addition, POx was studied for applications in sensors or biofuel cells [17–19], as a source for hydrogen peroxide to improve dough properties in baking [20], or as a biocatalyst for oxygen scavenging in single-molecule experiments [21]. Especially during the biocatalytic conversion of sugars it was noted that POx activity is rapidly lost while the enzyme is rather stable under noncatalytic conditions, *i*.*e*., in the absence of its carbohydrate substrate. Operational stability could be significantly improved by the addition of catalase to the reaction mixtures to remove hydrogen peroxide. However, even large excess of catalase could not stabilise POx activity completely under operational conditions [16,22,23]. Inactivation of POx during substrate turnover was studied and modelled in detail by Treitz *et al*. [24], and it was concluded that inactivation results from H_2_O_2_ or the hydroxyl radical derived from H_2_O_2_, which cause specific oxidation of certain amino acid residues. However, the exact molecular mechanism of inactivation was not revealed so far. It was the objective of our study to identify this mechanism of POx inactivation during turnover, and to identify amino acid residues that might be affected by the release of hydrogen peroxide during substrate turnover by POx.

## Material and Methods

### Chemicals and reagents

Dithiothreitol (DTT), α-cyano-4-hydroxycinnamic acid (CCA), trifluoroacetic acid (TFA) and 4-ethylmorpholine acetate were from Sigma (Steinheim, Germany) as were all other chemicals unless otherwise stated. Sequencing-grade trypsin was obtained from Promega (Madison, WI) and sequencing-grade Asp-N protease was from Roche Diagnostics (Mannheim, Germany). Peptide standards (angiotensin II, insulin B oxidised form) were from Bruker Daltonics (Bremen, Germany). MAGIC C18AQ chromatographic material was purchased from Michrom BioResources (Auburn, CA).

### Construction of POx Variants

Construction of the pET21d^+^/POx vector (pHL2), expressing a construct of the *T*. *multicolor pox* gene fused to a C-terminal His_6_ tag and placed under the control of the T7 promoter, was described earlier [11]. The plasmid pHL2 was used as template for all mutagenic PCRs. To obtain the variants F454A and F454Y, the forward primers F454A_fwd (5’-ccaccgcgatgctgccagttacggcgcag-3’), and F454Y_fwd (5’-atccaccgcgatgcttacagttacggc-3’) as well as the reverse primer F454A_rev/F454Y_rev (5’-agcatcgcggtggatctgagtgtg-3’) were used. The mutagenic PCRs were performed as follows: 98°C for 30 s, 30 cycles at 98°C for 10 s, 58°C for 20 s, 72°C for 4 min and 72°C for 7 min. PCRs were carried out using Phusion high-fidelity DNA polymerase from New England BioLabs (Ipswich, MA), deoxynucleoside triphosphates (dNTP) from Fermentas (St. Leon-Rot, Germany) and oligonucleotide primers from VBC Biotech (Vienna, Austria). PCR products were separated by agarose gel electrophoresis and purified using the Wizard SV Gel and PCR-Clean-Up System (Promega; Madison, WI). DNA was digested with *Dpn*I at 37°C for 2 h and purified. Plasmid DNA was transformed into electro-competent *E*. *coli* BL21 Star DE3 cells and after regeneration the cells were grown over night at 37°C on LB_amp_ plates (1% peptone, 0.5% yeast extract, 1% NaCl, 1.4% agar supplemented with 100 mg mL^-1^ ampicillin). The presence of the mutation of interest was verified by DNA sequencing (VBC Biotech) using the reverse primer T7_termrev (5’-gctagttattgctcagcgg-3’).

### Enzyme production and purification

Wild-type POx as well as the variants F454A and F454Y were produced and purified as reported [11]. In short, 2-L cultures of *E*. *coli* BL21 Star DE3 were grown in TB_amp_ medium in shaken flasks at 37°C and 160 rpm. After reaching an OD value at 600 nm of 0.5, gene expression was induced by adding 0.5% lactose. After incubation for further 20 h at 25°C and 160 rpm, cells were separated by centrifugation (4200×g, 30 min, 4°C). The cell pellet was resuspended in phosphate buffer (50 mM, pH 6.5) containing 1 g L^-1^ phenylmethylsulfonyl fluoride, and cells were broken up in a continuous homogenizer (APV Systems; Silkeborg, Denmark). The crude cell extract was obtained by ultracentrifugation (150,000×g for 30 min at 4°C), and one-step purification of the His-tagged proteins was done on 20 mL BioRad Profinity IMAC Ni-Charged Resin (Bio-Rad; Vienna, Austria). Enzymes were eluted with a linear gradient (50 mM Na phosphate buffer, 500 mM NaCl, 10–1000 mM imidazol, pH 6.5). The active fractions were concentrated and imidazole was removed by ultrafiltration using an Amicon Ultra Centrifugal Filter Device with a 30-kDa cut-off membrane (Millipore; Billerica, MA).

### Inactivation of *Tm*POx during glucose turnover or in the presence of H_2_O_2_

*Tm*POx (6 μM in 50 mM phosphate buffer, pH 6.5) was incubated under aerobic conditions with 100 mM D-glucose at 30°C. Alternatively, *Tm*POx (6 μM in 50 mM phosphate buffer, pH 6.5) was incubated with varying concentrations of H_2_O_2_ (0–500 mM) for 72 h. Samples (90 μL) were taken at different time points and the residual POx activity was measured. Prior to the standard ABTS activity assay 10 mg of MnO_2_ and 90 μL of a BSA stock solution (15 mg/mL) were added to each sample, this mixture was then incubated at 30°C and continuous shaking on an Eppendorf Thermomixer (300 rpm) for 10 min to destroy H_2_O_2_, which would interfere with the subsequent activity assay. MnO_2_ was removed by centrifugation before the enzyme assay.

### Enzyme assay and steady-state kinetic parameters

POx activity was measured spectrophotometrically at 30°C and pH 6.5 with the standard chromogenic ABTS [2,2’-azinobis(3-ethylbenzthiazolinesulfonic acid)] assay as described previously [25]. One Unit of POx activity was defined as the amount of enzyme needed for the oxidation of 2 μmol of ABTS per min under the assay conditions. Protein concentrations were determined by the Bradford assay using the BioRad Protein Assay Kit with BSA as standard. Kinetic studies were carried out at 30°C in phosphate buffer (pH 6.5) using the routine ABTS-peroxidase assay and oxygen (air) at saturation. The electron donor substrate D-glucose was varied in a range of 0.1–20 mM. The apparent steady-state kinetic constants were calculated from three independent measurements by non-linear least-square regression, fitting the data to the Henri-Michaelis-Menten equation, using SigmaPlot (Systat, Germany). Turnover numbers were calculated assuming a molecular mass of 68 kDa for the POx subunit.

### Preparation of oxidised POx and proteolytic digestion

Wild-type *Tm*POx was inactivated by the addition of 100 mM D-glucose or 500 mM H_2_O_2_. To this end, *Tm*POx (6 μM in 20 mM Bis-Tris buffer pH 7.0) was incubated for 4 h and 5 h, respectively, at 30°C. The incubation was stopped when the residual POx activity was approximately 5%, and the protein preparations were loaded onto a Mono-Q 5/5 anion exchange chromatography column (GE Healthcare) pre-equilibrated with 20 mM Bis-Tris buffer pH 7.0. The enzymes were eluted with a linear gradient of NaCl (0–1 M in the same buffer). SDS-PAGE was carried out on 10% gels according to Laemmli. The Coomassie Brilliant Blue-stained protein bands were excised from the gel, cut into small pieces and washed several times with 10 mM DTT, 100 mM 4-ethylmorpholine acetate (pH 8.1) in aqueous 50% acetonitrile (MeCN). After complete destaining, the excised gels were washed with water, shrunk by dehydration in MeCN, and again reswollen in water. The gels were then partly dried using a SpeedVac concentrator, and rehydrated with cleavage buffer containing 50 mM 4-ethylmorpholine acetate buffer pH 8.1, 10% MeCN, 0.01% mercaptoethanol and 1 μL of protease solution (50 ng/μL for trypsin and 10 ng/μL for Asp-N). After overnight digestion at 37°C the resulting peptide mixtures were acidified with 5% acetic acid.

### MALDI mass spectrometry

Positive-ion mass spectra were measured on a Bruker BIFLEX II time-of-flight mass spectrometer (Bruker; Bremen, Germany) equipped with a SCOUT 26 sample inlet and a 337 nm nitrogen laser (Laser Science; Cambridge, MA). The spectra of the peptides obtained after proteolytic digestion were measured in reflectron mode using CCA in aqueous 40% MeCN/0.2% TFA (10 mg/mL) as a MALDI matrix. One μL of sample was deposited on the MALDI target and after complete evaporation was overlaid with 1 μL of the matrix. The spectrometer was calibrated externally using the peptide standards angiotensin II and insulin B oxidised form with [M+H]^+^ ions of 1046.5 and 3494.6, respectively.

### μHPLC-nano ESI mass spectrometry

Peptide fragments were loaded onto a homemade capillary column (0.18 × 100 mm) packed with MAGIC C18AQ (5 μm, 200 Å) reversed-phase resin, and separated using a gradient from 5% MeCN/0.5% acetic acid to 50% MeCN/0.5% acetic acid for 60 min. The column was connected to an LCQ^DECA^ ion trap mass spectrometer (ThermoQuest; San Jose, CA) equipped with a nanoelectrospray ion source. The spectrometer was operating in MS/MS mode. MS/MS experiments were only performed on selected precursor ions corresponding to double and triple-charged ions of the peptide with oxidised Phe454. Tandem mass spectra were evaluated manually.

## Results and Discussion

### Stability measurements

**Fig 1:**
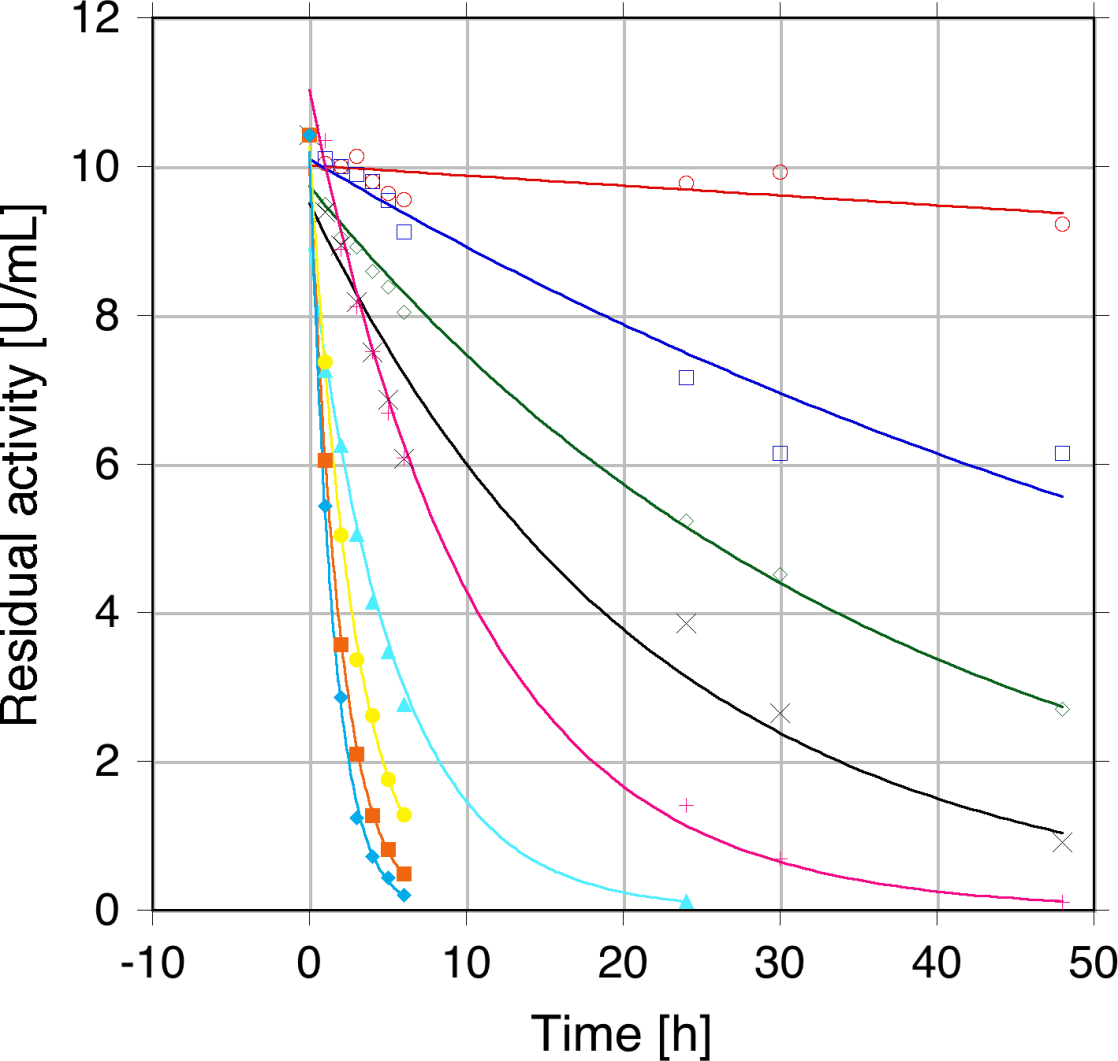
Inactivation of pyranose oxidase from *Trametes multicolor* (*Tm*POx) by hydrogen peroxide. *Tm*POx (6 μM in 50 mM phosphate buffer, pH 6.5) was incubated with varying concentrations of H_2_O_2_ and the residual POx activity was measured using the standard ABTS activity assay after exogenous H_2_O_2_ had been destroyed by MnO_2_. Symbols: ○ (red), 10 mM H_2_O_2_; □ (dark blue), 20 mM H_2_O_2_; ◇ (green), 50 mM H_2_O_2_; × (black), 100 mM H_2_O_2_; + (pink), 200 mM H_2_O_2_; ▴ (light blue), 300 mM H_2_O_2_; ■ (orange), 400 mM H_2_O_2_; ◆ (turquoise) 500 mM H_2_O_2_.

Pyranose 2-oxidase is a very stable enzyme under resting conditions, *i*.*e*., when it is not performing its catalytic reaction [4,24], with *Tm*POx for example showing a melting temperature of 60.7°C under non-catalytic conditions [26]. This is, however, in sharp contrast to stability under operational conditions. When *Tm*POx was incubated with 100 mM D-glucose under aerobic conditions (pH 6.5, 30°C) and in the absence of catalase, approximately half of the activity was lost within 90 min, and after 4 h of incubation only 6% of the initial activity was retained. It should be noted that D-glucose was still completely converted under these conditions despite this rapid decrease in POx activity. This decrease in activity presumably results from the formation of the second reaction product, hydrogen peroxide, or from reactive oxygen species (ROS) that are derived from H_2_O_2_. This was further corroborated by incubating *Tm*POx with varying concentrations of H_2_O_2_ (0–500 mM final concentration). Plots of residual activity versus time (Fig 1) showed that POx was inactivated by H_2_O_2_ in a time- and concentration-depending process following first-order kinetics. From these inactivation curves we calculated the half-life times of activity τ_½_, which decreased from 39.5 h at 10 mM H_2_O_2_ (pH 6.5 and 30°C) to 1.03 h at 500 mM H_2_O_2_ (additional τ_½_ values were 22.8, 12.5, 7.93, 2.96, 1.94 and 1.29 h at 20, 50, 100, 200, 300, 400 mM H_2_O_2_, respectively). The τ_½_ value for *Tm*POx activity was estimated to be >500 h under identical conditions (pH 6.5 and 30°C) when no hydrogen peroxide or D-glucose was added.

### Mass spectrometry

**Fig 2:**
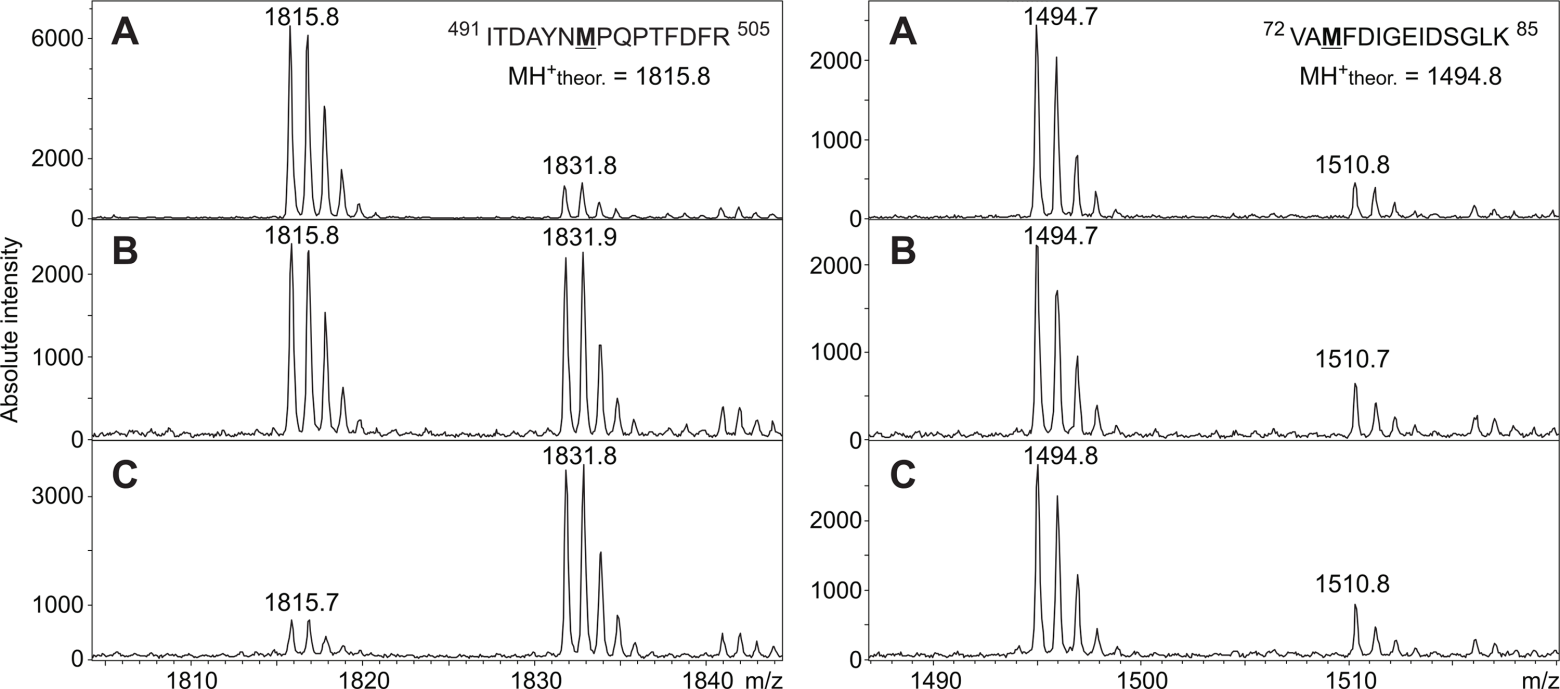
Mass spectrometric identification of methionine residues oxidized by H_2_O_2_ during *Tm*POx inactivation. MALDI MS spectra were measured for unaffected POx (**A**), for POx inactivated during D-glucose oxidation (**B**) or for POx inactivated by endogenous H_2_O_2_ (**C**). The selected MALDI spectra in the left panel illustrate that Met497 of the tryptic peptide ITDAYNMPQPTFDFR with a theoretical MH^+^ of 1815.8 was extensively oxidised in *Tm*POx inactivated either during substrate turnover (**B**, left panel) or by H_2_O_2_ treatment (**C**, left panel). In contrast, some methionine residues were found not to be oxidised during *Tm*POx inactivation as shown for Met74 of the peptide VAMFDIGEIDSGLK having a MH^+^ of 1494.8 (right panel). The small signals at m/z 1510.8 are related to the oxidized form of the peptide generated due to the presence of air oxygen.

**Fig 3:**
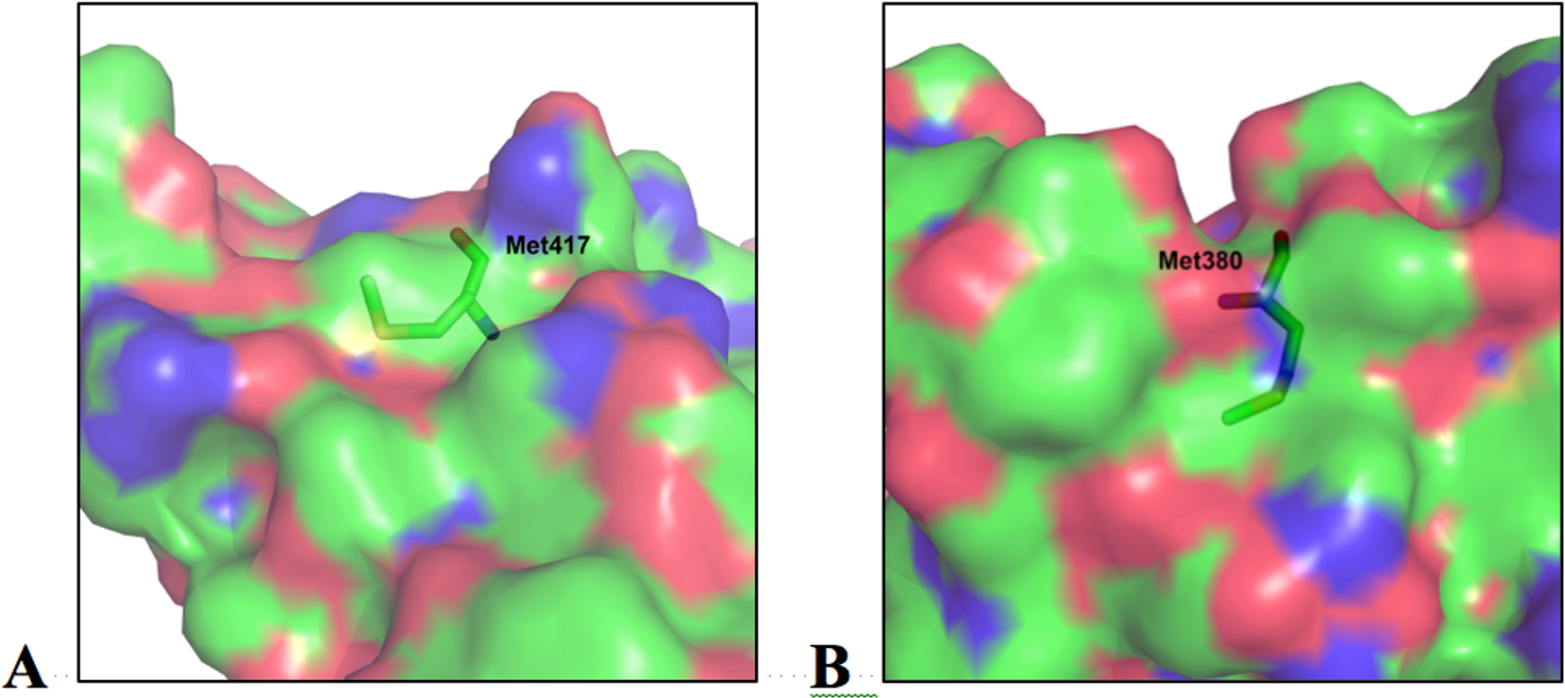
Accessibility of methionine residues. Surface of the *T*. *multicolor* POx monomer in the vicinity of (**A**) Met417, showing the surface-exposed sulphur atom which is oxidised by H_2_O_2_, and (**B**) Met380 with its sulphur-containing side chain pointing towards the interior of the polypeptide matrix, in which it is buried and hence is not accessible from the surface.

**Fig 4:**
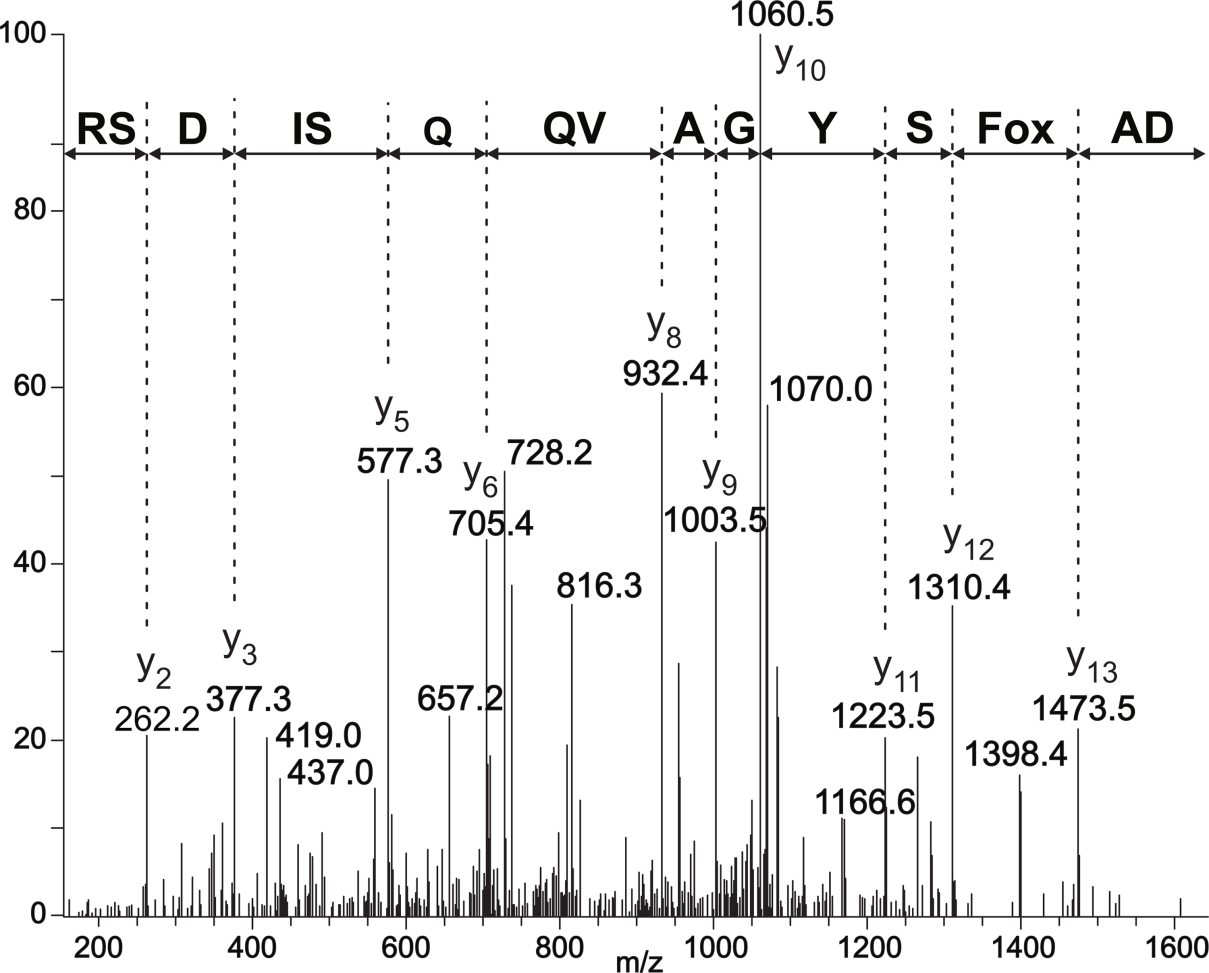
Identification of the exact site of oxidation in the peptide ^452^DAFSYGAVQQSIDSR^466^ by LC-ESI-MS/MS. The nearly complete series of C-terminal y-ions in the MS/MS spectrum of the double-charged ion of 830.4 confirms the peptide sequence. The +16 Da mass shift found for the ion y_13_ further indicates that Phe454 is oxidised during substrate turnover and by H_2_O_2_ treatment.

To investigate possible modifications of *Tm*POx during substrate turnover–either resulting from oxidation of susceptible amino acid side chains, or from Schiff bases formed between the ketosugar and the polypeptide–mass spectrometry was used. Both an enzyme preparation that was inactivated during D-glucose oxidation and a preparation inactivated by the addition of exogenous H_2_O_2_ were studied by MALDI-MS after enzyme digestion, and proteolytic fragments of these two preparations were compared with corresponding fragments of unaffected *Tm*POx, *i*.*e*., an enzyme that was not applied in substrate turnover experiments. Proteolytic digestion with trypsin and Asp-N gave together primary sequence coverage of approximately 70%, including a number of amino acids that are putatively accessible to oxidation. These experiments showed several fragments with a mass increase of 16 or 32 Da indicating oxidation of certain amino acids, while no indications were obtained that D-ketoglucose forms a covalent adduct to a peptide fragment under these reaction conditions. Most of these fragments showed a 16-Da increase to some extent even in the untreated enzyme sample, however, the intensity of this additional peak was only low, showing that oxidation occurs even in the presence of air oxygen or intracellularly in the presence of oxygen and sugar. This is an indication that mainly methionines are affected in these peptides since oxidation of Met residues by oxygen (as well as by H_2_O_2_) is well known [27]. These Met-containing peptides showed different behaviour in the enzyme samples that were inactivated during D-glucose turnover or by H_2_O_2_ treatment (Fig 2). In some of these peptides the intensity of the +16 Da peak increased considerably, which indicates that H_2_O_2_, either formed during the oxidation of D-glucose or added externally, oxidises these Met residues (Fig 2, left panel). This was observed for Met43 in the peptide of MH^+^ 887.5, Met497 in peptide MH^+^ 1815.8, Met555 in peptide MH^+^ 1720.9, or Met416 as well as Met417 in peptide MH^+^ 2139.9; this latter fragment was in fact the only one that showed mass increases of +16 and +32 Da, indicating that both Met residues are oxidised (Table 1). All of these affected methionine residues are located on the surface of the intact pyranose oxidase tetramer, their side chains are solvent exposed and will thus get in contact with H_2_O_2_ once it is released from the active site or added (Fig 3A). Some of these fragments showed a +16 Da mass increase, however, the intensity of this additional, small peak was not enhanced in the samples inactivated during sugar oxidation or by adding H_2_O_2_ compared to the untreated sample, which suggests that no oxidation by H_2_O_2_ occurs in addition to the slight oxidation by air oxygen, at least under the reaction conditions selected here (Fig 2, right panel). This was found for e.g., Met74 in peptides MH^+^ 1284.6 and 1494.6, or Met380 in peptides MH^+^ 722.4 and 2894.4 (Table 1). Met74 is part of the polypeptide core, internally buried and hence not solvent exposed, while Met380 is located at the surface of the protein, yet its side chain is buried within the polypeptide matrix and hence also not accessible (Fig 3B). In addition to the Met-containing fragments, MALDI-MS revealed peptide DAFSYGAVQQSIDSR with MH^+^ 1643.8 as being significantly oxidised by both catalytic turnover of D-glucose and addition of H_2_O_2_ forming a single-oxidised analogue with MH^+^ 1659.8. This fragment showed no indication of oxidation in the untreated sample, and furthermore it did not contain any of the usual suspects of oxidation by H_2_O_2_, *i*.*e*., the amino acids Met, Cys, Trp, or His [28,29]. In order to identify the exact site of oxidation this peptide was subsequently analysed by LC-ESI-MS/MS, which showed a +16 Da mass increase for Phe454, indicating that this phenylalanine residue is in fact oxidised both during substrate turnover and by endogenous hydrogen peroxide (Fig 4).

**Table 1:** Oxidation of methionines. Methionine-containing peptide fragments detected that show a mass increase of 16 or 32 Da after proteolytic digestion of *Tm*POx (inactivated during turnover of 100 mM D-glucose, treated with endogenous H_2_O_2_ or unaffected). Proteolytic digestion was performed with trypsin or Asp-N protease as indicated. The last column indicates whether the particular Met is considerably oxidised during substrate turnover and by H_2_O_2_ treatment compared to the unaffected sample (see also Fig 2).

Digestion	Peptide MH^+^ ion	Amino acids	Met position	Sequence	Oxidation
Trypsin	887.5	39–46	43	KVPGMDIK	Yes
Asp-N	1284.6	64–75	74	ELVGAGYKVAMF	No
Trypsin	1494.7	72–85	74	VAMFDIGEIDSGLK	No
Trypsin	722.4	378–383	380	SDMTIR	No
Asp-N	2894.4	379–405	380	DMTIRGTPGELTYSVTYTPGASTNKHP	No
Asp-N	2139.9	406–421	416, 417	DWWNEKVKNHMMQHQE	Yes, Yes
Trypsin	1815.8	491–505	497	ITDAYNMPQPTFDFR	Yes
Asp-N	1720.9	542–557	555	EPGLVLHLGGTHRMGF	Yes

### Kinetic studies

We prepared the variants F454A and F454Y by site-directed mutagenesis to evaluate the effect of modifications at this position on the reaction kinetics of *Tm*POx, and determined the apparent steady-state kinetic data for the oxidation of D-glucose in the presence of a constant concentration of oxygen at air saturation (Table 2). Oxidation of phenylalanine in polypeptides by ROS can yield a mixture of 2- and 3-hydroxyphenylalanine (*o*-, *m*-tyrosine) derivatives [30,31] and also the 4-hydroxyphenylalanine (*p*-tyrosine) derivative [32], and hence the Phe to Tyr substitution was selected, mimicking the proposed oxidation by hydrogen peroxide to some extent. Even though the Phe → Tyr substitution is conservative, it showed significant effects on the apparent turnover number *k*_cat_, which was reduced by approx. two-thirds compared to the value of recombinant wild-type POx for D-glucose oxidation (16.3 and 49.0 s^-1^, respectively; Table 2). Replacing Phe454 by alanine shows an even more dramatic effect and almost completely abolishes activity.

**Table 2:** Apparent steady-state kinetic constants of *T*. *multicolor* pyranose oxidase. Steady-state kinetic constants were determined for wild-type *Tm*POx and its variants with D-glucose (0.1–20 mM) as electron donor, and O_2_ (air) under saturation as electron acceptor.

	*k*_cat_ (s^-1^)	K_m_ (mM)	*k*_cat_/K_m_ (mM^-1^s^-1^)
wt POx^1^	49.0 ± 1.7	0.851 ± 0.140	57.6
F454A	0.479 ± 0.031	0.712 ± 0.011	1.47
F454Y	16.3 ± 0.3	0.948 ± 0.08	17.2

^1^ Recombinant POx from *T*. *multicolor* overexpressed in *E*. *coli* carrying a His_6_-tag

**Fig 5:**
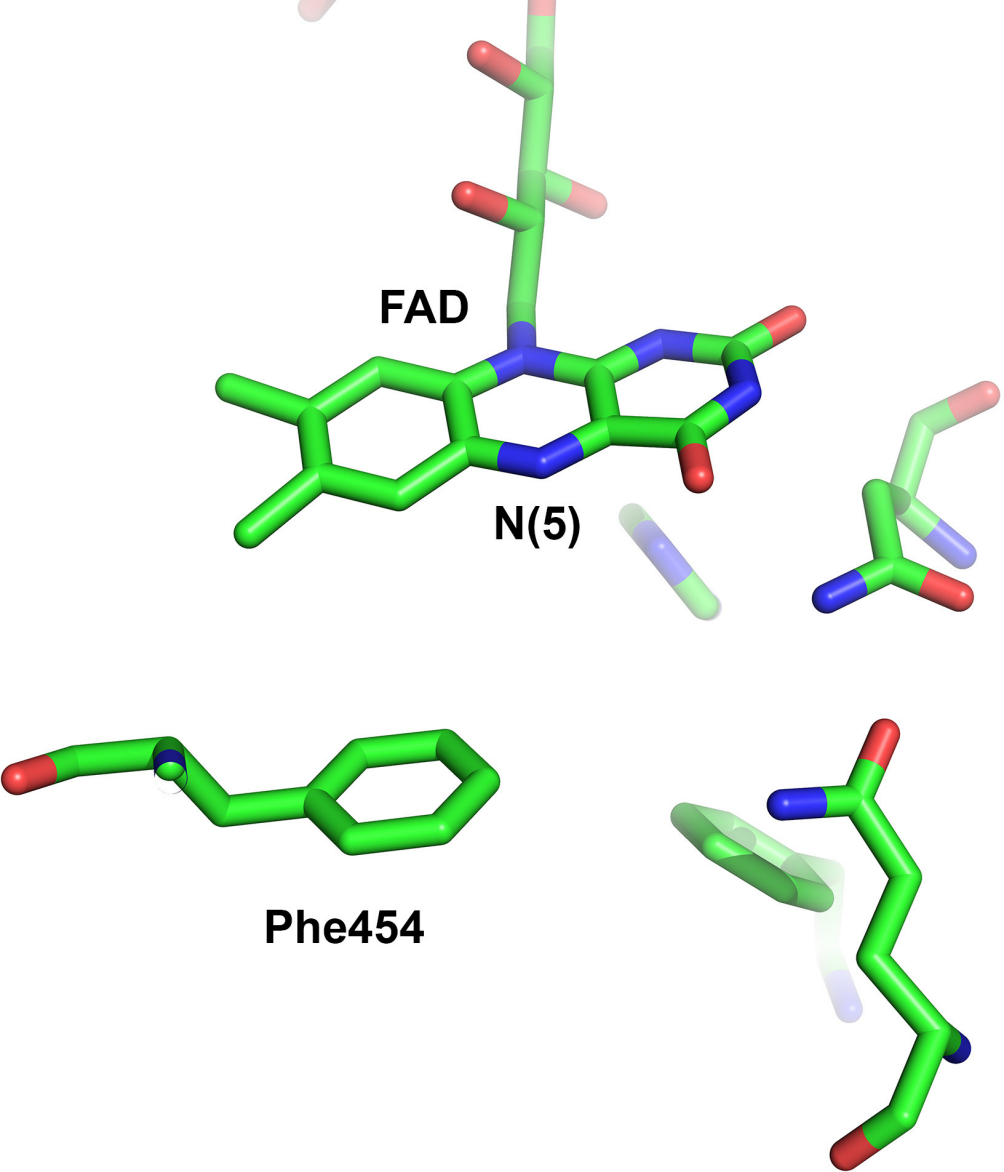
Active-site geometry of pyranose oxidase from *T*. *multicolor*. In the closed form of the active-site loop of pyranose oxidase from *T*. *multicolor* (*Tm*POx, PDB code 1TT0; [3]), which is thought to be relevant for the oxidative half-reaction of POx [3], Phe454 is positioned in the direct vicinity of the isoalloxazine ring and the C4a/N5 locus, at which oxygen is reduced. The figure was generated using PyMOL (http://www.pymol.org/).

Phe454 is located at an important position close to the isoalloxazine moiety of FAD (Fig 5), where oxygen is reduced to hydrogen peroxide [33]. The active site of *Tm*POx is gated by a conserved, highly flexible substrate-binding loop (residues 450–461), which displays substantial conformational degeneracy [3,11]. The gating segment (^454^FSY^456^) involves only the tip of this loop and appears to play a prominent role in determining the local environment and physicochemical characteristics of the active site, and thus is particularly important for discrimination of sugar substrates and for binding of oxygen. Furthermore, we recently showed that three conserved residues (Asp452, Phe454 and Tyr456) in this gating segment are essentially intolerant to substitution [12].

Phe454 is in direct vicinity of the reactive C4a-N5 locus of the isoalloxazine ring, where oxygen is reduced to hydrogen peroxide [14], and hence this residue will be exposed to H_2_O_2_ before it leaves the active-site cavity of POx. Phe454 is pointing towards the C4a-N5 locus of FAD in the closed conformation of the active-site loop, which is representing a state required for the binding of oxygen [11]. Phe454 is part of an elongated hydrophobic cavity, which is formed at the *re* side of the isoalloxazine ring upon closure of the substrate-binding loop and which is important for binding of oxygen and accommodation of a peroxide group at the C4a position [13]. Oxidation of Phe454 will change the hydrophobic character of this cavity, and might thus interfere with oxygen binding / peroxide accommodation in the active site of POx. We propose that oxidation of Phe454 is the first and main step in the inactivation of *Tm*POx by hydrogen peroxide. Oxidation of methionine residues might then further contribute to the complete inactivation.

## Abbreviations

ABTS: 2,2’-azinobis(3-ethylbenzthiazolinesulfonic acid)
BSA: bovine serum albumin
CBB: Coomassie Brilliant Blue
CCA: α-cyano-4-hydroxycinnamic acid
GMC family: glucose-methanol-choline family of oxidoreductases
DTT: dithiothreitol
IMAC: immobilised metal affinity chromatography
POx: pyranose oxidase
ROS: reactive oxygen species
TFA: trifluoroacetic acid
*Tm*POx: pyranose oxidase from *T*. *multicolor*
